# Case Reports: Management Challenges in Pediatric Germ Cell Tumors

**DOI:** 10.3389/fped.2021.659083

**Published:** 2021-04-15

**Authors:** Sophie Carr, Arash Safavi, Erik D. Skarsgard

**Affiliations:** ^1^Department of Surgery, BC Children's Hospital, University of British Columbia, Vancouver, BC, Canada; ^2^Faculty of Medicine, University of British Columbia, Vancouver, BC, Canada; ^3^Department of Child Health and Surgery, University of Arizona College of Medicine, Phoenix, AZ, United States

**Keywords:** germ cell tumor, teratoma, malignant, alpha-fetoprotein, recurrence, retroperitoneal teratoma, surgery

## Abstract

Germ cell tumors in infants are most frequently extragonadal, benign, and amenable to surgical resection. An unusual feature of germ cell tumors is the potential coexistence of malignant with benign disease which makes it possible for patients with incompletely resected tumors to experience either a benign or malignant recurrence. A challenge to postoperative surveillance is the interpretation of serum alpha fetoprotein, a marker of malignancy, that is physiologically elevated during the first year of life. A rare subset of germ cell tumors occur in the retroperitoneum. Although the vast majority are benign, these tumors are often large and distort normal anatomy, and may demonstrate local invasiveness that increases risk of resection. The intent of these reports is to caution readers about these unusual features of germ cell tumors of infancy.

## Introduction

Germ cell tumors (GCT), often referred to as teratomas account for ~3% of neoplasms in children under 15 years (1). Among infants, more than 80% of GCT are benign, with the commonest location being sacrococcygeal (2). Retroperitoneal GCT account for <4% of all GCT and tend to present within the first year of life (3).

Malignant GCT elaborate alpha fetoprotein (AFP), which serves as a marker of malignancy, but can presents challenges in interpretation during the first year of life when serum AFP levels are physiologically elevated. Pathologic assessment of resected tumors must take into account the potential for coexistence of benign with malignant histology and underscores the importance of postoperative AFP surveillance as a marker for residual or recurrent malignant tumor.

For localized tumors with benign histology, primary resection is curative. Although most benign teratomas are encapsulated and usually amenable to complete extracapsular resection, retroperitoneal teratomas (RT) have a tendency to cause anatomic distortion and infiltration of vital structures including the abdominal aorta, its visceral branches, and the inferior vena cava.

We present three cases which highlight: (i) pitfalls in postoperative AFP surveillance leading to a delay in detection of a malignant recurrence after resection of a benign sacrococcygeal tumor (SCT); and (ii) challenges in the surgical management of RT which emphasize the importance of preoperative imaging and intraoperative risk-assessment in balancing treatment goals with surgical safety.

## Case Descriptions

### Case 1

A healthy term female underwent a spinal ultrasound on day of life 3 because of asymmetry in her intergluteal cleft. The ultrasound identified several small cysts adjacent to the coccyx, consistent with a small cystic SCT. Spinal magnetic resonance imaging (MRI) confirmed a 12 mm × 10 mm × 26 mm multiloculated cystic lesion in the pre- and post-coccygeal space. Serum AFP was measured to be 150 ug/L on day of life 104 which was appropriate for normal physiologic elevation at 3 months of age (4), while beta human chorionic gonadotropin (β-hCG) was normal at <2.0 IU/L.

**Figure 1:**
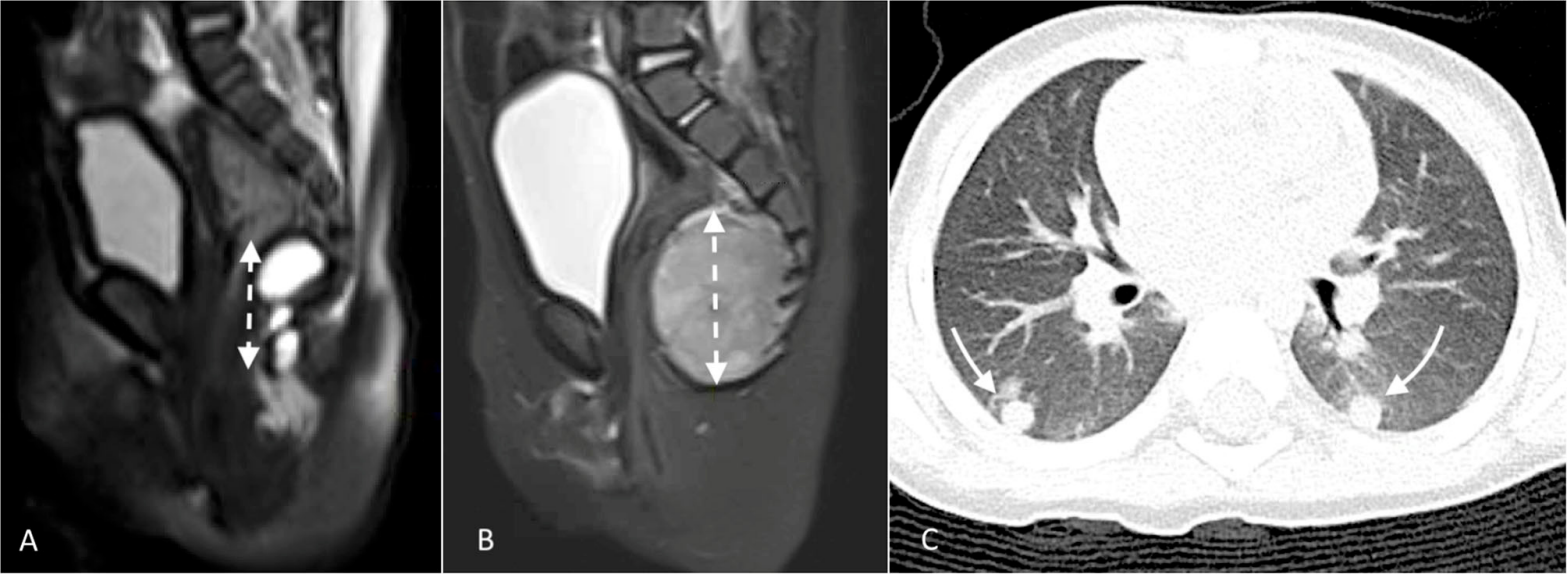
Select images from Case 1: **(A)** Initial MRI demonstrating lobulated cystic mass which on pathologic exam post-resection was mature cystic teratoma; **(B)** MRI 8 months later demonstrating pre-sacral, solid tumor recurrence; and **(C)** CT scan showing simultaneous, bilateral lung metastases which biopsy confirmed to be malignant teratoma (yolk sac tumor).

The child underwent excision of the cystic mass with partial coccygectomy. One of the cysts was adherent to the rectal wall and was ruptured during dissection. The pathology was reported as mature cystic teratoma, completely resected. A serum AFP was not repeated until 8 months later and at that time had risen to 7,425 ug/L, β-hCG was <1.0 IU/L. Abdominal pelvic MRI revealed a 4.2 cm × 3.5 cm × 3.0 cm solid sacrococcygeal mass, and chest computed tomography (CT) demonstrated bilateral pulmonary metastases (Figure 1). A video-assisted thoracoscopic surgery (VATS) biopsy of the lung lesions confirmed metastatic, malignant GCT with yolk sac differentiation. The patient was treated with 4 cycles of cisplatin, etoposide, and bleomycin with near complete normalization of AFP (12 ug/L), resolution of metastatic lung nodules, and cytoreduction of the sacrococcygeal mass to 1.5 cm × 1.9 cm × 2.9 cm. She underwent complete resection of the mass with completion coccygectomy, and pathology showed necrosis with no viable yolk sac tumor identified. Postoperatively, two further cycles of chemotherapy were completed. Her serum AFP remained slightly elevated for a short period post treatment but has since normalized at 6.3 ug/L. She remains clinically and radiologically free of recurrent disease at 33 months.

### Case 2

**Figure 2:**
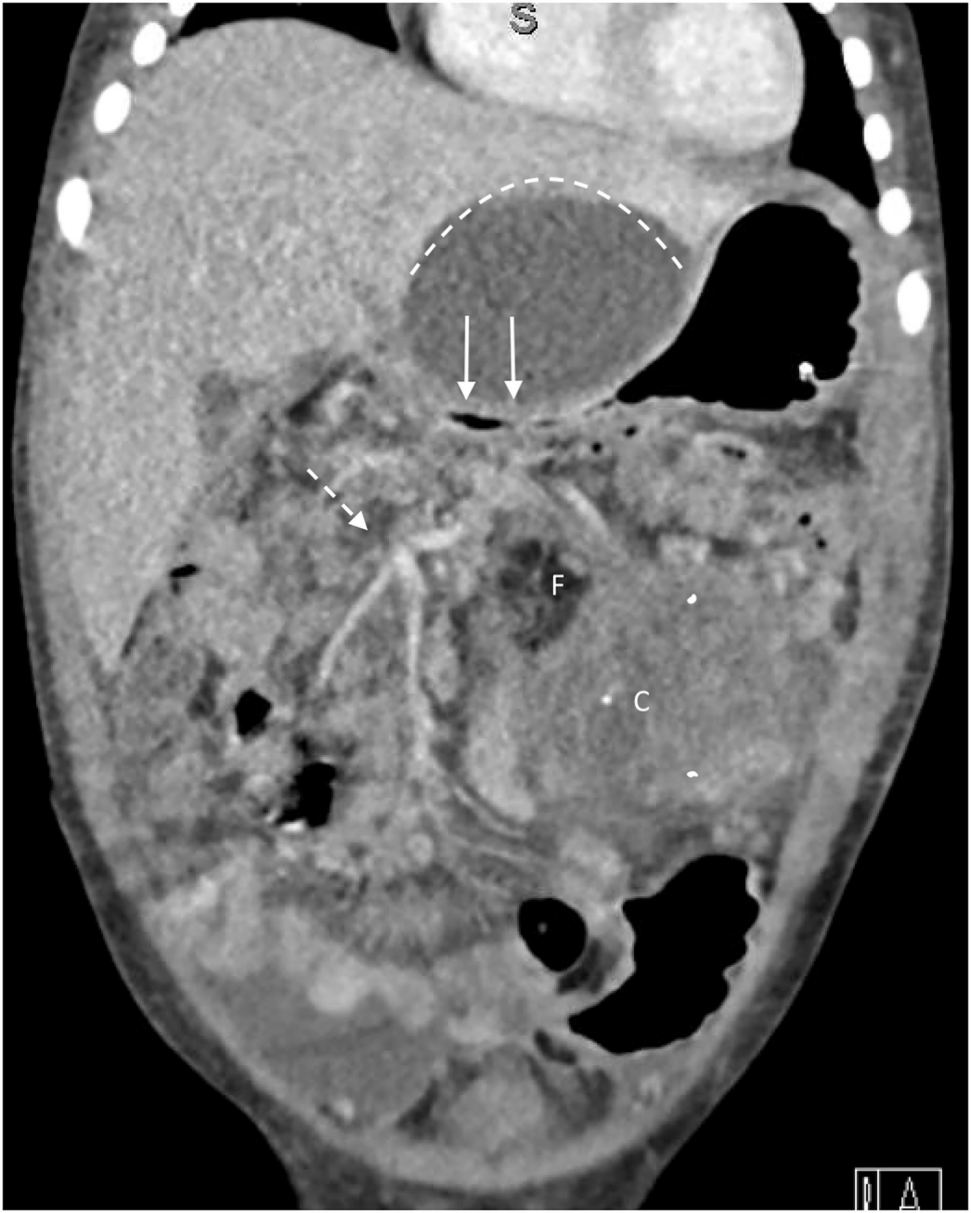
Coronal section of CT scan from Case 2 demonstrates superior limit of cystic component (dotted line) which is compressing duodenum causing gastric outlet obstruction (solid arrows). The tumor contains fat (F) and areas of calcification (C), and encases superior mesenteric artery branches to the transverse colon (dotted arrow).

A 20 month-old female presented to hospital with abdominal pain and features of gastric outlet obstruction. Abdominal and pelvic CT scan delineated a 11.4 cm × 8.0 cm × 7.7 cm, mixed solid and cystic retroperitoneal mass obstructing the gastric outlet with areas of calcification and fat, consistent with a mature teratoma (Figure 2). AFP was mildly elevated at 25 ug/L, while β-hCG was within normal range at < 1 IU/L. The patient was discussed at our multidisciplinary oncology conference where the consensus diagnosis was mature teratoma based on its radiologic appearance and normal tumor markers, despite the fact that it had some invasive features, and a decision to proceed with resection was made. Esophagogastroduodenoscopy showed significant amounts of bilious fluid in the stomach but no evidence of gross tumor invasion into the duodenum. An exploratory laparotomy was undertaken which revealed tumor infiltration of surrounding structures, including the duodenum and transverse colon mesentery. Multiple core and incisional biopsies were taken, and intraoperative and subsequent permanent section analyses supported a diagnosis of mature teratoma with no immature or malignant elements.

Given the invasive nature of this apparently benign tumor, two cycles of carboplatin, etoposide, and bleomycin, were given to see if any cytoreduction could be achieved. Post-chemotherapy imaging revealed no change in the areas of invasion with slight overall enlargement of the tumor.

Magnetic resonance cholangiopancreatography demonstrated a margin of normal duodenum below the Ampulla of Vater and above the site of duodenal invasion by tumor. A second laparotomy was undertaken with a goal of complete tumor resection. The tumor capsule was densely adherent to the duodenum, inferior mesenteric artery, left renal hilum, and aorta. A predominantly extracapsular tumor resection included the 3rd and 4th parts of the duodenum (beginning approximately 2 cm distal to the Ampulla), mesentery of the transverse colon, and the inferior mesenteric artery near its origin. The dilated 2nd part of duodenum was anastomosed end to end to the proximal jejunum, and the transverse and descending colon were resected, leaving an ascending colostomy and distal Hartmann pouch. Procedural blood loss was 500 cc and an intraoperative transfusion was required. The child returned to the operating room 2 weeks later for duodenojejunal anastomotic revision, and then several months later for colostomy closure. Pathological examination of the tumor was that of mature teratoma without immature elements, with a positive epithelial margin noted on the posterior aspect. She remains free of recurrent disease at 24 month clinical and radiological follow-up.

### Case 3

**Figure 3:**
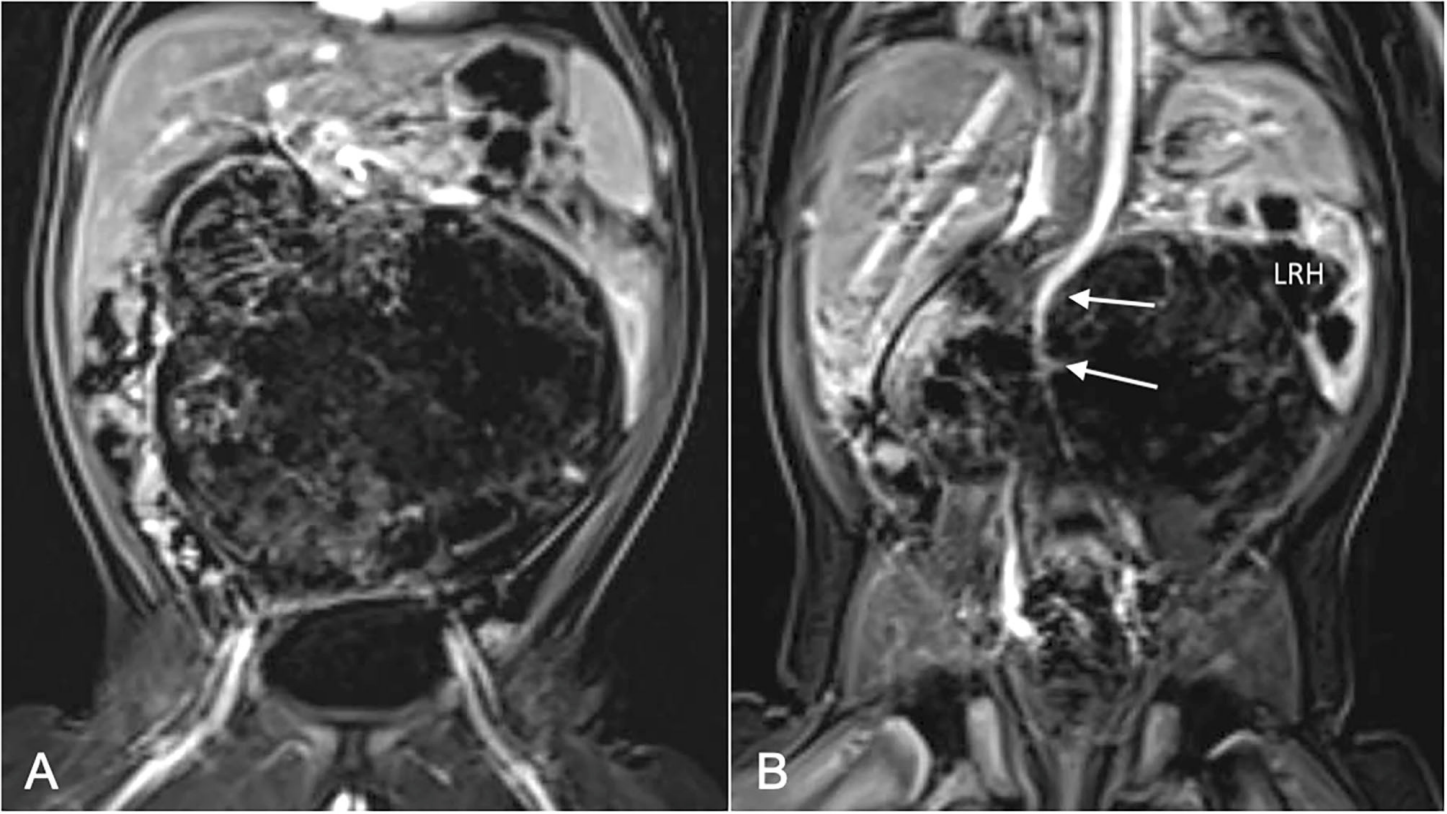
MRI images from Case 3: **(A)** Coronal section at level of iliofemoral vessels shows large, laterally encapsulated retroperitoneal tumor; **(B)** Coronal section posterior to **(A)** shows deformation of abdominal aorta (solid arrows) by tumor, and infiltration of left renal hilum (LRH) causing hydronephrosis.

A previously healthy 2 month-old male presented to hospital with a large abdominal mass and 1 week history of non-bilious emesis. Abdominal MRI demonstrated a 11.3 cm × 10.5 cm × 8.1 cm, mixed solid-cystic, encapsulated retroperitoneal mass (Figure 3). The tumor effaced the abdominal aorta below the superior mesenteric artery takeoff, as well as the common iliac arteries, flattening and displacing them to the right. The inferior vena cava (IVC) was similarly compressed with complete effacement of most of its retroperitoneal course. The left kidney was displaced laterally and superiorly with a moderate hydronephrosis from mass effect on the proximal left ureter. A chest CT scan revealed multiple small nodules of indeterminate significance. Serum AFP was significantly elevated at 36,000 ug/L, while β-hCG was normal at < 1 IU/L. Case discussion at our multidisciplinary oncology conference considered the option of preoperative chemotherapy, however based on imaging features and apparent tumor encapsulation the prevailing opinion was that this tumor was most likely an immature teratoma with foci of malignant GCT, and despite its size, was likely amenable to primary resection.

The patient underwent bilateral ureteral stent placement followed by an exploratory laparotomy which revealed a large, partially encapsulated tumor. The tumor was adherent to the mesentery of the transverse and descending colon, and superiorly to the root of the small bowel mesentery, but these attachments were carefully dissected exposing the anterior aspect of the tumor. Posteriorly the tumor was adherent to the aorta and IVC, and it became clear that a complete extracapsular dissection would not be feasible or safe. Notably, the tumor encased the left proximal ureter and was densely adherent to the left renal artery and vein. Following tumor resection, efforts were made to go back and excise areas of remnant capsule. The patient received a transfusion for an estimated intraoperative blood loss of ~300 cc.

Pathology on the resected specimen was predominantly immature teratoma, grade 2–3, with focal yolk sac tumor. There was a positive left retroperitoneal margin (immature teratoma) corresponding to areas of adherence to the left kidney and ureter. Two weeks after surgical resection, his AFP had dropped to 3,000 ug/L, and it continued to decrease thereafter, reaching a normal range within 2 months of surgery.

Three months after surgery, a recurrence in the left retroperitoneum was noted on follow-up ultrasound and confirmed on MRI. Serum AFP remained within normal limits for his age. The patient was taken back to the operating room for excision of recurrent tumor which was again encasing the left ureter and hilar vessels, and infiltrating the descending colon mesentery, requiring a left nephrectomy and partial left hemicolectomy with a primary anastomosis. Pathology on the resected recurrence was that of mature teratoma with no immature or yolk sac elements. At 15 month follow-up, he remains clinically and radiologically disease-free.

## Timeline

**Figure 4:**
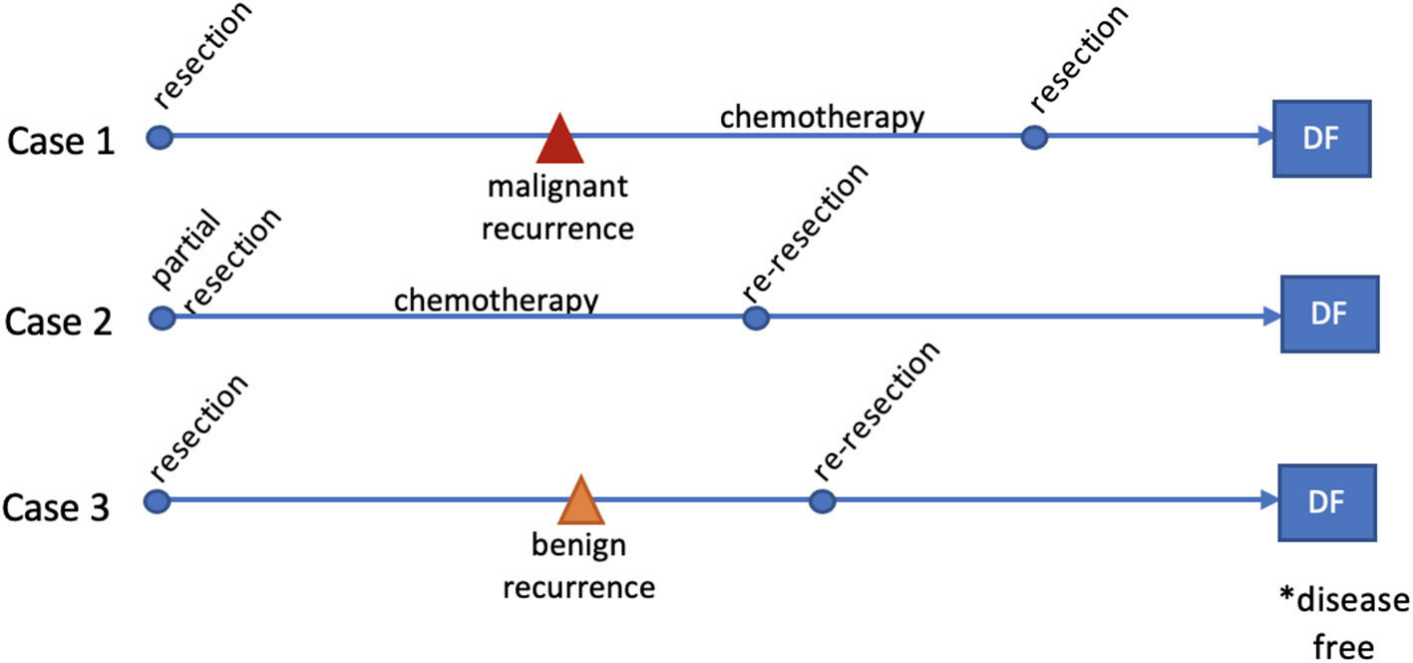
Timelines for cases 1–3 summarizing treatment, recurrences and final outcome.

The treatment sequence, intermediate and final outcomes for each case are summarized in Figure 4.

## Discussion

Germ cell tumors arise from molecular aberrations in early germline progenitors known as primordial germ cells. These cells migrate from the yolk sac along the gonadal ridge in the midline of the developing embryo. Disruption of the migration process is the likely explanation for the midline propensity of extragonadal GCTs. The pluripotent nature of these cells accounts for the range in observed histology. Immature teratomas contain less differentiated tissue, including neuroectoderm, and are assigned a grade (I-3) according to the degree of dedifferentiation. Although mature and immature teratomas are benign tumors, the need for thorough histologic evaluation of immature teratomas, particularly those with high grade features, is important given the known association with microscopic foci of malignancy (5).

### Post-resection Surveillance of SCT

There are several factors which increase the likelihood of SCT recurrence after resection, including incomplete resection, failure to remove the coccyx and tumor rupture/spillage during surgery (6). Because mature SCTs are benign, it is tempting to assume that a malignant recurrence is impossible, and that follow-up should emphasize local recurrence surveillance. Our experience with case 1 demonstrates the error of that assumption. Even though the resected specimen consisted of mature cystic histology and was reported to be completely resected, the patient recurred with metastatic yolk sac tumor within 8 months. We assume that the original tumor was incompletely excised, leaving behind a focus of malignancy that was sufficiently small so as to not elevate the serum AFP above its physiological level (150 ug/L at 3 months of age). Alternatively, the residual tumor remaining after surgery was benign, but underwent malignant transformation.

A recently published meta-analysis of 22 studies identified a total of 999 patients with mature histology following SCT resection, from which there were 102 recurrences (10.2%). Of these, 57 (56%) proved to be malignant recurrences, and almost all were yolk sac tumor (7). There is an even higher association of malignancy with immature teratoma as confirmed by a review of 135 pediatric cases of immature teratoma, of which 75 (56%) were noted to be mixed tumors at diagnosis, and another 15 were identified following pathologic re-review of tumors with an initial diagnosis of immature teratoma (8). Other studies have confirmed the phenomenon of missed foci of malignancy in both mature and immature teratomas with a malignant recurrence, which underscores the importance of systematic tumor assessment by a pathologist with expertise in GCT interpretation (9, 10).

Recently published guidelines for follow-up surveillance of resected SCT with benign (mature or immature) histology emphasize the importance of physical examination, selective imaging and serum AFP monitoring until at least 3 years after initial treatment (11). Following GCT resection in any newborn or infant, AFP monitoring during infancy should be monthly to ensure that an early recurrence is not confused with a persistent physiologic AFP elevation. Table 1 summarizes a recommended 3 year schedule for surveillance following GCT resection in infancy.

**Table 1:** Recommended follow-up schedule following germ cell tumor resection.

	**Monthly**	**3 monthly**	**12 m**	**15 m**	**18 m**	**21 m**	**24 m**	**27 m**	**30 m**	**33 m**	**36 m**
AFP/ßHCG	+		+	+	+	+	+	+	+	+	+
Clinical Exam		+	+	+	+	+	+		+		+
Primary site Imaging		+	+		+		+				+
Chest X-ray			+				+				

### Surgical Management of Retroperitoneal Teratomas

These rare tumors usually present as an abdominal mass in infancy, although they can also be diagnosed prenatally (12). Preoperative cross-sectional imaging with CT or MRI reveals the typical findings of a large, well-defined mass that is predominantly solid with cystic areas, fat and calcification. Delineation of vascular anatomy, particularly visceral arterial branches is critical and may necessitate CT angiography or spiral CT with 3D reconstruction, so that areas of potential vascular compression, displacement or encasement are known preoperatively (13). Our two cases exemplify these distinguishing features of RT. The findings of direct invasion into the wall of the duodenum and mesentery of the colon in case 2 led to attempts to prove malignancy with intraoperative biopsies (despite only a mildly elevated serum AFP) and ultimately resulted in unnecessary chemotherapy and delayed resection. Case 3 highlights the challenges of intraoperative decision-making in determining extent of resection. The decision to preserve the kidney during the initial laparotomy in the presence of tumor infiltrating the hilum and encasing the proximal ureter almost certainly contributed to a local recurrence that ultimately required nephrectomy.

Other reports describe similar challenges in the surgical management of RT. Jones and Burns describe 6 children (5 infants) with benign RT that displaced and/or enveloped the aorta and IVC with infiltration of the mesentery and adjacent viscera (14). They report intraoperative bleeding, which in one case required IVC ligation, and visceral injuries including gastric and common bile duct injuries. One patient died 12 days postoperatively from complications of massive intraoperative bleeding and sepsis. Another series of 14 patients with benign RT were treated at a median age of 5.5 months (15). Although complete resection was achieved in 13 patients, morbidity was significant in 6, including renal ischemia, common bile duct injury, diaphragm injury and vascular injuries including a portal vein injury. Qureshi et al. reported 15 patients (9 mature and 6 immature teratomas), of whom 10 underwent surgery in infancy (16). Two patients with large midline immature teratomas extending into the root of the mesentery underwent partial resection and chemotherapy without appreciable benefit. Other significant complications included massive blood loss and need for nephrectomy and partial gastric and colon resection.

In summary, we report three cases of GCT (2 in infants) which highlight some of the unusual biologic tendencies of these rare tumors. An awareness of the estimated 10% frequency of malignant recurrence of presumed benign GCT underscores the importance of early and frequent postoperative tumor marker surveillance to ensure timely detection of recurrence. An appreciation of the propensity for RT to be locally invasive despite their benign histology, should inform preoperative preparation and guide intraoperative decision-making. Finally, pre- and post-operative multidisciplinary discussion of these unusual tumors increases the likelihood of care optimization.

## Data Availability Statement

The raw data supporting the conclusions of this article will be made available by the authors, without undue reservation.

## Ethics Statement

Ethical review and approval was not required for the study on human participants in accordance with the local legislation and institutional requirements. Written informed consent to participate in this study was provided by the participants' legal guardian/next of kin. Written informed consent was obtained from the minor(s)' legal guardian/next of kin for the publication of any potentially identifiable images or data included in this article.

## Author Contributions

All authors listed have made a substantial, direct and intellectual contribution to the work, and approved it for publication.

## Conflict of Interest

The authors declare that the research was conducted in the absence of any commercial or financial relationships that could be construed as a potential conflict of interest.
